# Data and knowledge management in translational research: implementation of the eTRIKS platform for the IMI OncoTrack consortium

**DOI:** 10.1186/s12859-019-2748-y

**Published:** 2019-04-01

**Authors:** Wei Gu, Reha Yildirimman, Emmanuel Van der Stuyft, Denny Verbeeck, Sascha Herzinger, Venkata Satagopam, Adriano Barbosa-Silva, Reinhard Schneider, Bodo Lange, Hans Lehrach, Yike Guo, David Henderson, Anthony Rowe, on behalf of the IMI OncoTrack and the IMI eTRIKS consortia

**Affiliations:** 1 Luxembourg Centre for Systems Biomedicine, University of Luxembourg, Esch-sur-Alzette, Luxembourg; 2 Alacris Theranostics GmbH, Berlin, Germany; 3 Janssen Pharmaceutica NV, Beerse, Belgium; 4 Max Planck Institute for Molecular Genetics, Berlin, Germany; 5 Dahlem Centre for Genome Research and Medical Systems Biology, Berlin, Germany; 6 Data Science Institute, Imperial College London, London, UK; 7 Bayer AG, Berlin, Germany; 8 Janssen Research and Development Ltd, High Wycombe, UK

**Keywords:** Translational medicine, Data management, Oncology, Precision medicine

## Abstract

**Background:**

For large international research consortia, such as those funded by the European Union’s Horizon 2020 programme or the Innovative Medicines Initiative, good data coordination practices and tools are essential for the successful collection, organization and analysis of the resulting data. Research consortia are attempting ever more ambitious science to better understand disease, by leveraging technologies such as whole genome sequencing, proteomics, patient-derived biological models and computer-based systems biology simulations.

**Results:**

The IMI eTRIKS consortium is charged with the task of developing an integrated knowledge management platform capable of supporting the complexity of the data generated by such research programmes. In this paper, using the example of the OncoTrack consortium, we describe a typical use case in translational medicine. The tranSMART knowledge management platform was implemented to support data from observational clinical cohorts, drug response data from cell culture models and drug response data from mouse xenograft tumour models. The high dimensional (omics) data from the molecular analyses of the corresponding biological materials were linked to these collections, so that users could browse and analyse these to derive candidate biomarkers.

**Conclusions:**

In all these steps, data mapping, linking and preparation are handled automatically by the tranSMART integration platform. Therefore, researchers without specialist data handling skills can focus directly on the scientific questions, without spending undue effort on processing the data and data integration, which are otherwise a burden and the most time-consuming part of translational research data analysis.

**Electronic supplementary material:**

The online version of this article (10.1186/s12859-019-2748-y) contains supplementary material, which is available to authorized users.

## Background

The data coordination activities of large multi-stakeholder research collaborations are becoming more complex. Increasingly, projects are citing the use of specialist knowledge management technologies such as the tranSMART platform [1] as used by the IMI UBIOPRED, ABIRISK and OncoTrack projects [2–5]. In reality, however, a knowledge management platform alone is not sufficient to provide the tools to support all of the data management and coordination tasks to enable a consortium to gain the maximum value from its data. Without a data coordination platform that not only provides a common point of access for the accumulated data sets, but also allows a seamless transfer to analytical tools, the effective exchange of data, ideas and expertise is compromised, which devalues the data and delays the progress of the project.

The motivation to improve such technologies is therefore twofold: Firstly, the system provides a single place where data from all partners participating in the project can be deposited, collated, linked and then published back to the whole consortium. Secondly, the data are not just made *available* in curated form, but are also made *accessible.* This is achieved by the use of flexible user interfaces, combined with analytical and visualization tools that can be used by all stakeholders in the consortium and not just those with the specialist data handling skills such as bioinformaticians and statisticians. A consortium that provides a data coordination capability accelerates the work of the specialist data scientist who can access the raw data from a single location for specialist analysis. If this data coordination capability additionally includes a knowledge management technology, this can empower the wider community of scientists who are able to browse and generate hypotheses from all of the data in an accessible format.

In this paper, we present the broad overall systems architecture developed by the eTRIKS consortium to accommodate the data management requirements of translational research consortia, using the IMI OncoTrack project as a use case. Additionally, we present a novel plug-in for tranSMART developed by the IMI eTRIKS consortium to overcome some of the limitations in cross-linking related datasets, such as those found when exploring and conducting correlation analyses using clinical data, experimental data from patient derived ex vivo models and high dimensional “omics” data. The data linking solution presented here is capable of handling and integrating the majority of data types encountered in translational medicine research, independent of the medical indication, and should therefore be generally useful for other consortia faced with similar data management challenges.

In line with the challenges and requirements mentioned above, this knowledge management platform intends to provide a common point to access and share the accumulated, curated and pre-processed data sets as well as testing hypotheses and facilitating exchange of ideas.

The intended users and usages are:

1. All “end-users” that do not necessarily have advanced IT skills to be able to explore the integrated datasets with dynamic visual-analytics to test new hypotheses immediately, without asking bioinformaticians for every (explorative) analysis.
2. Bioinformaticians to select and download data (curated or raw) for specific analyses.
3. Data managers as well as researchers to collect, organise, store and disseminate data during the course of the project.
4. Project managers to oversee project progress in terms of available data and metadata.

We would like to emphasis that the analytical tools provided on the platform are not meant to replace all advanced analyses that might be carried out by trained bioinformaticians and biostatisticians, who nevertheless can benefit from the reduced time and effort needed for data preparation.

## Implementation

### The IMI OncoTrack consortium

The IMI OncoTrack Consortium [3] is an ambitious international consortium that is focused on advancing “Methods for systematic next generation oncology biomarker development”. As one of the Innovative Medicines Initiative (IMI) oncology projects, it brings together academic and industry scientists from more than twenty partner institutions in a research project to develop and assess novel approaches for identification of new markers for the treatment response of colon cancer.

At the core of OncoTrack are two patient cohorts that, either prospectively at the point of primary colon cancer surgery or retrospectively at the point of metastasis surgery are sampled in order to build a colon cancer tissue bank containing both primary and metastatic tumour samples, together with associated normal tissues and biofluids. A part of each tissue sample is also used to develop in vitro 3D cell cultures and in vivo xenograft models that are used to study response to standard and experimental therapies.

The tissue samples are processed to build collections of DNA, RNA, serum and circulating tumour cells that are then analysed to generate an in-depth description of the genome, transcriptome, methylome and proteome both of the tumour and the biological models. This approach uses a broad panel of methods such as next generation sequencing, proximity extension assays, reverse phase protein arrays, methylation arrays and mass spectrometry. The patient-derived models also provide platforms to study the role of tumour progenitor or ‘cancer stem cells’ in the pathogenesis and evolution of colon cancers.

Finally, data from all of these platforms are combined using a systems biology approach that can be used to make personalised predictions about how an individual may respond to therapy. The systems biology model of the cancer cell incorporates the combined results of genome, transcriptome, methylome and proteome analyses [6].

**Fig. 1:**
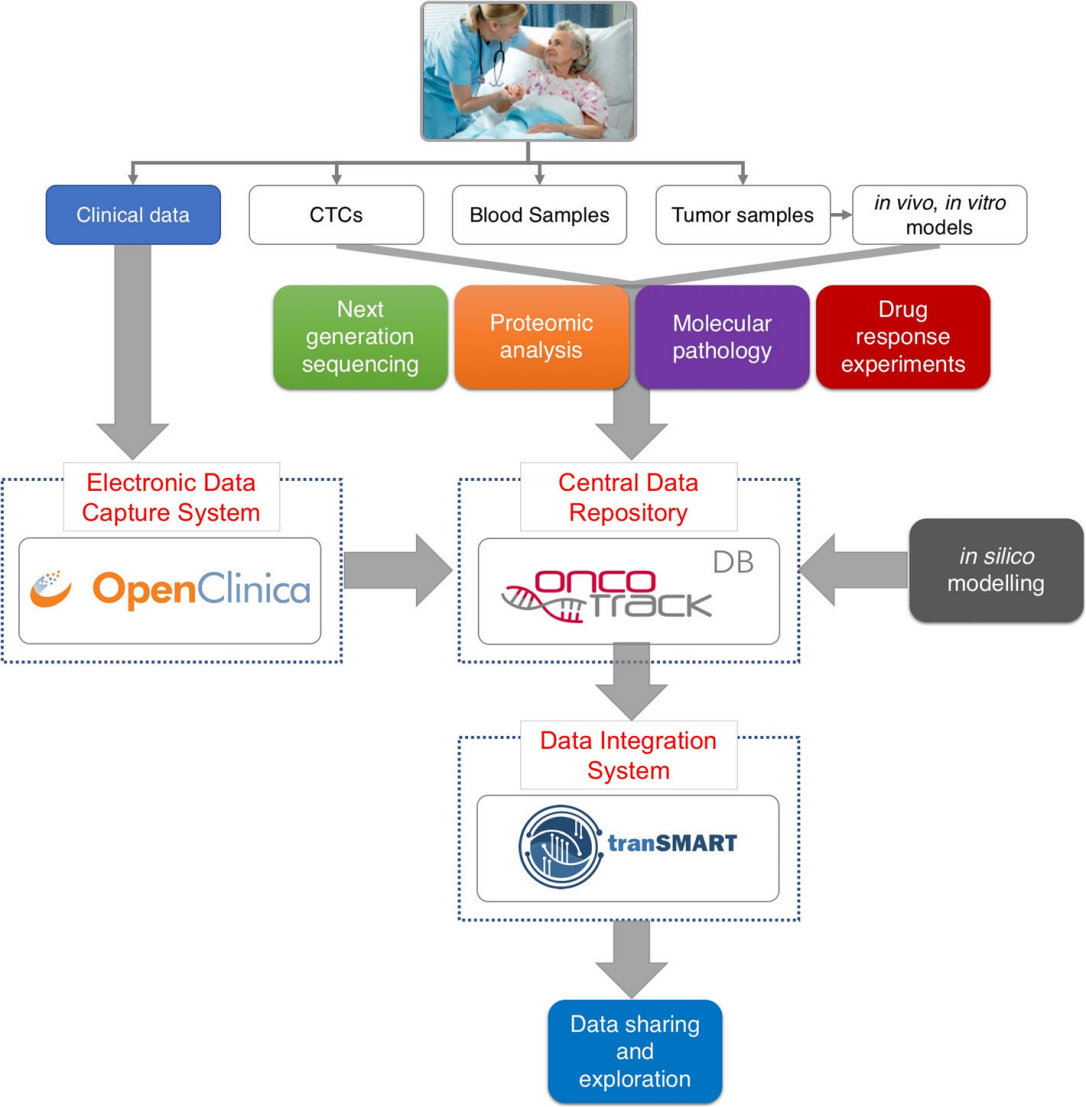
The components of the OncoTrack data coordination operation. The platform comprises three major components: the Electronic Data Capture System (EDC, OpenClinica), the Central Data Repository (OncoTrack DB), and the Data Integration System (tranSMART). The OpenClinica EDC system is used to collect medical history and observational patient data from clinical sites during the studies and feeds the structured data to the Central Data Repository. The Central Data Repository, OncoTrack DB is a sample indexed content management system. Data and results generated in the laboratories (before integration) are deposited and exchanged here. In order to link the different data types and layers, the data collected in the OncoTrack DB are integrated in the Data Integration System, tranSMART. The tranSMART data warehouse provides deep linking and integration between the clinical and laboratory data and a set of tools for the exploratory analysis of the integrated data

The coordination of these different collections of data requires core systems to be used to perform the data collection and integration tasks. We would like to note that the “data integration” related to the work reported here are the steps and procedures to transform and store data from subject level, sample level and derived animal models as well as across different data types (drug response, different molecular and ‘omics data) in an interlinked manner in a data warehouse. In this way users are able to filter data in any layer/type and query related data in the same or different layer/type with a few mouse clicks and subsequently test their new hypotheses. As shown in Fig. 1 and detailed below, the OncoTrack data management work package implemented OpenClinica [7] and developed the OncoTrack DB [8] as central repositories for clinical and biological data, respectively. Here, we describe the collaborative effort to interface these data repositories with tranSMART, to provide an interactive user interface for exploration and preliminary data analysis.

### OpenClinica: electronic data capture Fig. 1

The first component of the data coordination platform is the OpenClinica Electronic Data Capture system (EDC, https://www.openclinica.com/; https://github.com/OpenClinica/OpenClinica). OpenClinica provides the capability for the clinical sites to record electronically all of the patient data from different visits and to deposit these in a central database. The system enables the design of specific data entry conventions and data validation checks. These features ensure high data quality by providing all clinical sites with identical case report forms and by flagging data entry errors so they can be rapidly fixed. The user interface is made available through a standard web browser technology so that it requires no installation of software, allowing it to be readily adopted by all clinical sites. In order to ensure data privacy and compliance with data protection legislation, access to OpenClinica is IP-restricted and each clinical site can access only to the data for their own patients. In compliance with the institutional ethics committee and patient data privacy regulations, only a subset of the clinical data is made available to all consortium scientists through OncoTrack DB.

### OncoTrack DB: sample indexed content management

The Oncotrack DB is software based on DIPSBC (data integration platform for systems biology collaborations), further developed by Alacris Theranostics and adapted to the specific needs of the OncoTrack project [8]. It is best described as a “Sample Indexed” Content Management System (CMS). It supports the typical features of a CMS to store, version control and manage collections of files and also enables project management, dissemination and progress tracking as well as allowing multiple channels for data access (eg. web interface, RESTful API). File formats were developed to store the results of the different laboratory analyses including the NGS based genome and transcriptome analysis, the ex vivo drug response experiments and the molecular characterisation of tumour samples. For each experimental data type, a unique upload interface was deployed to handle specific requirements with regard to data production frequency, volume and format as well as transfer method (i.e. web interface, RESTful API). Additionally, the OncoTrack DB indexes each of these data files with unique sample identifiers, so that each file can easily be filtered to locate and sort all data by cohort, experimental platform or patient. Throughout this work, we have adopted generally accepted data standards for ‘omics, clinical data etc. where applicable, inter alia CDISC compliant terminology for clinical data using Study Data Tabulation Model (SDTM), high-throughput sequencing data standards (e.g. FASTQ, BAM), gene sequence variations data format (VCF) or Systems Biology Markup Language (SBML) for computational models. In addition, data was loaded into a relational database and mapped to respective reference standards (e.g. Ensembl, UniProt, miRBase) to allow comparability and ensure compatibility. This allowed for more advanced data access and querying of available data sets.

### tranSMART: knowledge management data warehouse

To make the data collected in OpenClinica and the OncoTrack DB accessible to the entire consortium in a systematic way, the tranSMART knowledge management platform was used. tranSMART is an open-source data warehouse designed to store data from clinical trials, as well as data from pre-clinical research, so that these can be interrogated together in translational research projects. tranSMART is a web-based system, designed for use by multiple users, across organizations. Prior to uploading data into tranSMART, a curation step (to adapt formats and define the data tree) needs to be performed. The data pre-processing is handled during this curation phase and ensures that the end-user is presented with data sets upon which valid hypotheses can be based. To ensure data integrity, it is recommended that the pre-processing and uploading be restricted to a limited group of data curators, working with uniform ETL scripts (https://github.com/transmart/tranSMART-ETL).

**Fig. 2:**
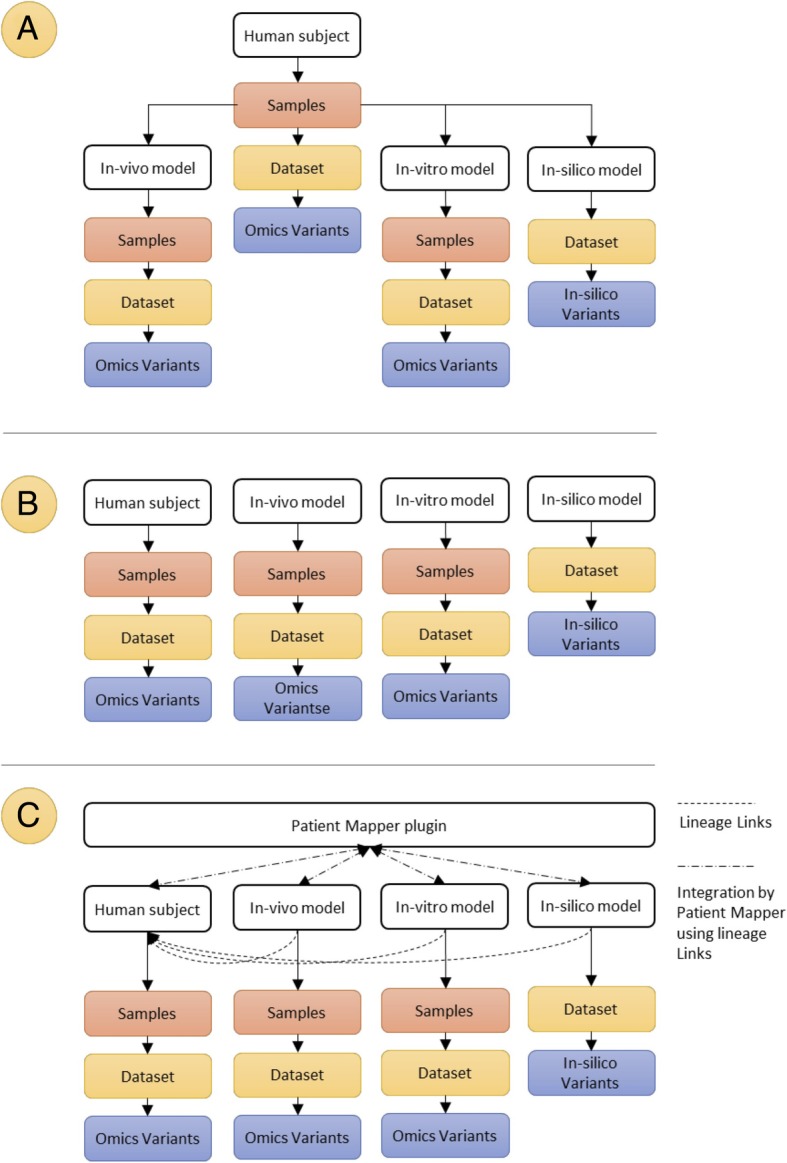
The OncoTrack dataset structure. **a** The complex OncoTrack data hierarchy with OMICS datasets directly generated from patient material and datasets generated from patient derived pre-clinical in vivo*,* in vitro and in silico models. **b** Due to constraints in tranSMART (v16.1) unable to represent this hierarchical use of samples, data has been organised as a series of different independent collections. One collection for data derived directly from patient samples and other collections for data derived from the pre-clinical models. **c** A solution we provided with linkage back to human subject and a tool to automatically map data using this linkage

The data were organised in 3 core collections: 1) the observational clinical cohorts, 2) the drug response data from the cell-line models and 3) the drug response data from the xenograft models (see Fig. 2). The high dimensional data from the molecular analyses were linked to these collections so that users could browse and analyse:

- Variants among germline, primary and metastatic tumour material
- Confirmatory genomic analyses of xenograft and cell cultures
- Quantification of RNA transcripts from clinical and preclinical samples
- Quantification of small non-coding RNA (miRNA)
- Analysis of DNA Methylation

The implementations of the functions reported in this manuscript have been integrated into the tranSMART main release, starting with version 16.2 (https://wiki.transmartfoundation.org/pages/viewpage.action?pageId=10126184). The code can be accessed under:

- https://github.com/transmart/transmartApp and https://github.com/transmart/SmartR
- The documentation can be found at: https://transmart-app.readthedocs.io/en/latest/
- A description of and link to a public demonstration version of the tranSMART instance can be found at https://wgu.pages.uni.lu/etriks-oncotrack/

### Dynamic dataset linking

The Oncotrack consortium based its approach to biomarker discovery on the innovative experimental design of creating collections of patient derived pre-clinical models. Tumour tissue collected during surgery from both the primary and metastatic tumours was used to create in vitro 3D-cell line models and xenograft in vivo models that could be linked back to the original patient. Cell lines and xenografts were used to study the response to a standard panel of established and experimental colon cancer drugs. The combination of deep molecular characterization of the tumours and their associated models with data on drug response provides the scientist with the necessary information for identification of candidate biomarkers for prediction of response to treatment.

Data generated in the OncoTrack study is organised so that each sample can be linked back to the patient from whose tissue it was generated, as shown in Fig. 2a.

The primary data level is the human cohort, with the primary entity being the subject. Patient tissue samples collected from subjects are profiled using omics and NGS technologies creating datasets directly attributable to the subject. A second data level is generated from the three disease modelling platforms used by OncoTrack: xenograft based in vivo models, 3D cell line based in vitro models (‘biological models’) and cell simulation based in silico models. Each of these is used to explore the tumour samples in different experiments such as response to standard clinical or novel experimental therapies. The biological models are then profiled using NGS and omics analysis technology, generating their own dataset and variants. The primary entity of this data is the model used in the experiment (e.g. cell line) with a lineage to the original patient. This two level lineage hierarchy of the datasets is shown conceptually in Fig. 2a.

This approach contrasts with the data model of tranSMART that has (by design) been developed with constraints regarding data organization. These constraints are required in order to achieve the required interactions of a flexible data model to a suite of analysis tools. These constraints mean that when modelled in tranSMART the data has to be modelled as 4 independent data sets (Fig. 2b) or coerced to a structure resembling Fig. 2a but at the loss of being able to use the analysis and visualisation tools.

Our objective was to create a mechanism where 1) data sets could be analysed independently *and* 2) we were able to respect the lineage of the samples to enable integrated analysis between the different levels in the hierarchy in the dataset. Our solution, shown in Fig. 2c is to maintain the basic tranSMART structure shown in Fig. 2b, augmented with additional metadata about lineage, mapping all level two datasets to their “parent” in the cohort dataset.

Additionally, we developed PatientMapper, a plugin-tool for tranSMART designed to integrate data sets from different levels of the hierarchy referring to these mapped lineage relationship metadata. When applied across datasets with the lineage mapping, Patient Mapper uses the back-links to correctly integrate and reshape the data to be compatible with the tranSMART analytics suite.

### Data curation for dynamic data linking

To support dynamic data-linking among datasets, we developed an enhanced curation process to create a data model that includes lineage relationships between different entities. To achieve this, we developed a new mapping logic, in which the parent-child relationships are kept for all levels of datasets to the patient from which the samples/derived model are derived (see Fig. 2c). For example: a patient is a parent of *n* patient samples. Those samples can again be a parent of *m* in vitro models (like e.g. xenografts or xenograft treatment groups). Those in turn can be parents of *p* samples used for ‘omics measurements, or even of ‘child’ in vitro models, etc.)

**Fig. 3:**
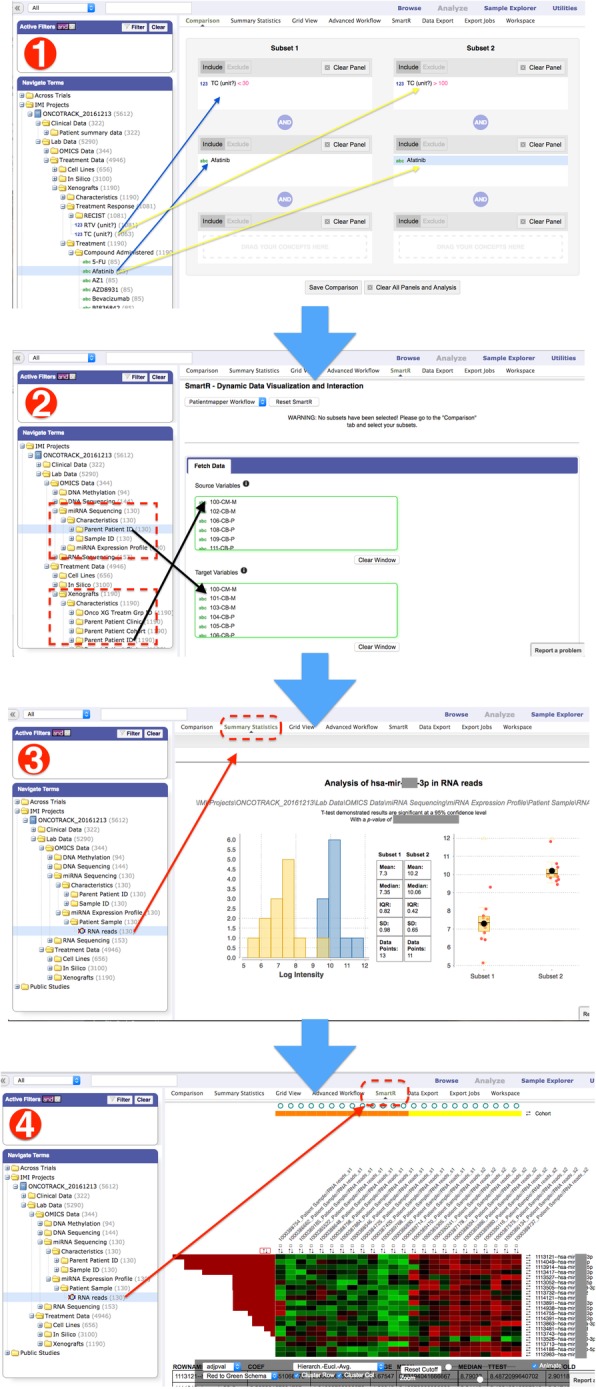
Integration of OncoTrack data into tranSMART: (1) Left panel: Overall data representation in the TranSMART data tree. Right panel: easy customized cohort building with drag-and-drop. (2) Cascaded querying with cohort linking/selection tool PatientMapper. (3) Generating summary statistics of a miRNA of choice by dragging the miRNA-Seq node to the right panel and providing miRNA ID using the HiDome plugin. (4) Performing miRNA-ome wide heatmap analysis between the two sub-cohorts (here responder vs. non-responder for a selected drug treatment) using SmartR workflows

In tranSMART, variables are represented in a tree structure (i2b2 tree, see Fig. 3 and see also Additional file 1) [9]. The design of the data tree structure should organise the data to allow easy exploration of datasets. In line with the above considerations, in the OncoTrack-tranSMART integration, we separated different data levels and data types into separate study-trees to better organise the different categories (clinical data and lab data). Under the Clinical Data tree, general subject information (e.g. Clinical site, Cohort, etc.) of the participating subject are stored. The Lab Data stores data generated in the lab (e.g. Treatment Data, OMICS Data). In each subtree under the “Treatment Data” and the “OMICS Data”, the subject/sample information as well as the interrelationships to other subtrees are organized in the “Characteristics”, and the corresponding measured data are stored within the subtree labelled with the data type (e.g. Xenografts, DNA_Methylation, etc.)

Data curation and transformation are a prerequisite for the implementation of the data model described above. These steps are sometimes time consuming and require detailed knowledge regarding the necessary pre-processing of each data type as well as familiarity with tranSMART ETL requirements and scripting skills. Within the work reported in this paper, however, the curation need only be performed once and periodic updates (while new data of the same data type are generated) can be done automatically with pipelines developed during the manual curation. Data contributed by the different partners contributing to OncoTrack were collected centrally in OncoTrack DB. To avoid the risk of variability in the process, curation and transformation were performed centrally using one uniform set of ETL scripts. Details of each curation step are described in the Additional file 1.

### Dynamic cross-layer data link tool (PatientMapper)

One typical query/analysis that requires the above-mentioned data model could be: what are the differences between xenograft models that respond to a certain drug and those that do not respond to the same drug: how do their parent samples differ in transcriptome and/or epigenome? To enable users to easily explore such a data model with dynamic cross-layer data, we have developed a user-friendly data linking tool (PatientMapper. see Fig. 3 (2)) that allows users to easily link sub-cohorts they have built on any level of data to datasets in other levels for the corresponding parent/children sample/subjects. This tool is integrated into tranSMART and updates cohort selection automatically based on the linking parameters selected by the user. From this point on, the other analysis and exploration of the updated cohorts can be performed within the same platform. This tool is not limited to mapping sample level data to patient level data but can be used to map data across any levels as long as they share a common lineage.

### Results visualization

High Dimensional and Omics Exploration (HiDome) is a novel functionality for tranSMART that was developed through eTRIKS Labs [10]. It extends the platform’s core capabilities with regard to handling omics data. HiDome allows the visualization of individual components of these data sets, for example the read count distribution for a given miRNA (see panel 3 in Fig. 3). It also enables creation of cohorts based on omics data set components, for instance comparing patients with a high versus a low read count for a specific miRNA. Details about the development of HiDome are described in a separate paper [11].

SmartR is another new functionality for tranSMART that was also developed through eTRIKS Labs [12]. This functional module enables the user of tranSMART to perform interactive visual analytics for translational research data, including both low-dimensional clinical/phenotypic data and high-dimensional OMICS data (see panel 4 in Fig. 3).

## Results

### Oncotrack TranSMART

**Fig. 4:**
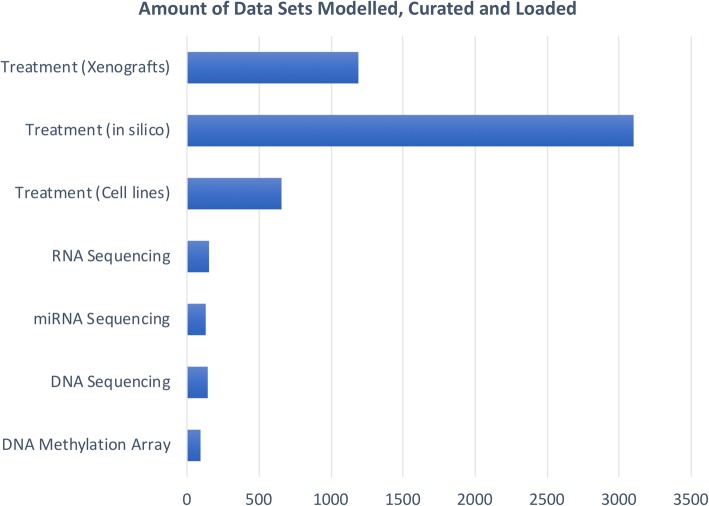
An overview of OncoTrack data that have been modelled, curated and loaded in the OncoTrack tranSMART Server

The current Oncotrack TranSMART deployed to the consortium is based on the eTRIKS distribution (eTRIKS V3) of tranSMART 16.1. A summary of data that have been modelled, curated and loaded in the OncoTrack tranSMART server is shown in Fig. 4.

### Case study

To illustrate how the OncoTrack TranSMART can facilitate the exploration and analysis of data, we present here the use case already introduced in the discussion of the PatientMapper (see above). We would like to emphasise that this paper is not meant to focus on any specific scientific questions within the OncoTrack project, which have been reported in a separate paper [13], but rather to demonstrate the advantage of the tranSMART platform in solving data integration problems in general. For this reason, the marker annotations are blanked out.

The use case: For two xenograft groups, one whose tumours respond to treatment with Afatinib, the other one whose tumours are resistant, what biomarkers (e.g. miRNA) are different in their parent patient tumor samples? And how to check whether a marker of interest is differentially presented?

The steps: Researchers who use the OncoTrack-tranSMART can achieve this goal easily by first building the two cohorts (xenografts Afatinib responders vs xenografts Afatinib non-responders) by dragging the Afatinib data-node and treatment response TC values (with filters, here < 30 and > 100) from the data tree into cohort selection (See Fig. 3 (1) for details). In order to get the miRNA data of the corresponding source patient, users can link the cohorts that were built using the xenograft level data to patient level data (here: miRNA sequencing data) using the GUI tool PatientMapper (Fig. 3 (2)) that will automatically handle the many-to-one relationship across the different data layers. In this example, the patient level miRNA expression profile (from miRNA-Seq) is linked to the xenograft level treatment response data by simply dragging-and-dropping their Parent Patient ID branch on the i2b2 tree to the PatientMapper tool.

With this new cohort after data mapping, researchers can easily check and visualize the corresponding miRNA sequencing data between the two sub-cohorts via the Summary Statistics function in tranSMART, by dragging the miRNA sequencing data node into it (See Fig. 3 (3)).

Researchers can extend the same steps to analyze the differences across the complete miRNA data set, using a few mouse-clicks to run the SmartR workflow (Fig. 3 (4)) to explore and identify differential biomarkers between the responders and non-responders. In all these steps, data mapping, linking and preparation are handled automatically by the OncoTrack-tranSMART integration platform. Therefore, researchers can focus directly on the scientific questions, without spending any effort on processing the data and data-integration, which is otherwise a burden and the most time-consuming part of translational research data analysis.

## Discussion

### Data platforms for translational medicine and cross-omics integration

Recent reviews have summarized many of the existing computing and analytical software packages designed to ease integrated analysis of ‘omics and/or clinical data [14–16]. Those platforms are either repositories with an existing infrastructure or solutions requiring deployment. The advantage of the first type of solutions is their out-of-the-box usability, but this sacrifices the flexibility of configuration and toolset management. This type is represented by technologies like STRIDE [17], iDASH [18], caGRID and its follow up, TRIAD [19, 20] or BDDS Center [21]. Many platforms in this category focus on a specific disease, like cBioPortal [22] or G-DOC [23, 24] for cancer, or COPD Knowledge Base [25] for pulmonary dysfunction. The second family of solutions requires deployment on the user’s infrastructure, often requiring substantial storage or High-Performance Computing (HPC) capabilities, but allows more flexibility in the setup and easier development. As a result of their configurable nature, such solutions provide support to ongoing projects as (part of) their data management platform to handle complex data. Examples in this group are BRISK [26], tranSMART [1] or Transmed [27]. Informative use cases of such platforms are SHRINE [28] and DARiS [29], where well-defined demands of clinical research projects drove the design and implementation of infrastructure supporting translational medicine.

Besides these platforms, there are also many solutions that target web-based integrated analysis of ‘omics data. Some well-known examples are EuPathDB (a eukaryotic pathogen genomics database resource, [30]), the DNA Microarray Inter-omics Analysis Platform [31], Mayday SeaSight (combined analysis of deep sequencing and microarray data, [32]), GeneTrail2 (multi-omics enrichment analysis, [33]), OmicsAnalyzer (a Cytoscape plug-in suite for modeling ‘omics data, [34]), PathVisioRPC (visualise and analyse data on pathways, [35]), 3Omics (analysis, integration and visualization of human transcriptomic, proteomic and metabolomic data, [36]) and PaintOmics (joint visualization of transcriptomics and metabolomics data, [37]).

Among the above-mentioned solutions, tranSMART stands out as a community-driven, rapidly growing, web-based data and visual-analytics platform for clinical and translational research [1, 16]. TranSMART is being used by many (> 100) organizations and consortia around the world [2–5, 16, 38–40]. It enables the integrated storage of translational data (clinical and ‘omics) by providing interlinks between different data-types and it allows researchers to interactively explore data as well as to develop, test and refine their hypotheses. These features are essential in order to support multi-party consortia like OncoTrack, that involve researchers with very diverse background working together on the datasets generated during the project. In the eTRIKS consortium, the platform has been further developed to incorporate more advanced, user-friendly and portable functionalities [40–44].

This paper describes the approach used by eTRIKS to provide an interface between the data architecture in the OncoTrack consortium and tranSMART. We also highlight the development of a new plug-in for the tranSMART platform to support dynamic data-linking among different datasets and datatypes in tranSMART.

The consortium model approach to research problems is becoming increasingly successful, as seen by the continuation of the European Innovative Medicines Initiative and the similar programs such as CPATH and the Accelerated Medicines Partnerships in the USA. There is increasing awareness among both funding agencies and the coordinators of large consortia, that data coordination and knowledge management capabilities are prerequisites for data to be integrated and used by all stakeholders in the collaboration and therefore constitute a key part of a project’s operational design. Developing a strong data coordination capability enables:

- Project Coordinators to understand the progress of data generation by different laboratories within the project, to help manage the scientific deliverables of a project and to identify in an early stage any data quality problems
- Clinical and Laboratory scientists, as by interacting with a knowledge management platform they have access to all of the data from across the consortium, not just the sections they generated themselves
- Data Scientists, Bioinformaticians and Statisticians to have access to clean, curated and linked datasets that represent the master version of data, saving them time in performing their own data preparation

While there are significant advantages to the investment in such a capability it should be recognised that there is no gold standard for data and knowledge management. As we have shown here, 3 key components (Open Clinica, OncoTrack DB, tranSMART) are used to collect, organise, publish and support analysis of the data generated in the OncoTrack consortium. While all of the software is Open Source and does not require a license for its implementation, there are operational costs in both the underlying IT hardware and the multi-disciplinary skill sets of people acting as data coordinator.

## Conclusions

The authors suggest that results generated from exploratory analysis as described here provide a useful approach to hypothesis generation, but that such results should be scrutinized by a qualified statistician or bioinformatician prior to publication.

During the course of OncoTrack, we were confronted by the reality of the maxim “Scientific research and data production in life sciences move faster than development of the technical infrastructure”. We developed patient derived pre-clinical models on a large scale and amassed large data sets from the analysis both of these models as well as the biological characteristics of the clinical samples. Consequently, new technology had to be developed to support the dynamic data linking across different datasets to enable the users to formulate the queries and analyses they wanted to explore. The approach described here is generally applicable to data collected in typical translational medicine research projects.

## Availability and requirements

Project home page: e.g. https://oncotrack.etriks.org

Project name: e.g. Oncotrack-eTRIKS data and knowledge management platform

Operating system(s): Linux

Programming language: Grail, javascript, R

Other requirements: Tomcat7, JDK 7, Postgres 9.3 or higher

License: tranSMART is licensed through GPL 3. SmartR is licensed through Apache.

## Additional file

Additional file 1: Supplementary Materials. (DOCX 26 kb)

## Abbreviations

CMS: Content Management System
DB: Data base
EDC: Electronic Data Capture
IMI: Innovative Medicines Initiative
