# Supplementary material for: Data and knowledge management in translational research: implementation of the eTRIKS platform for the IMI OncoTrack consortium
Source: BMC Bioinformatics. 2019 Apr 1;20:164. doi: 10.1186/s12859-019-2748-y (PMC6444691; doi:10.1186/s12859-019-2748-y)
Supplement: Supplementary file 1 — Supplementary Materials. (DOCX 26 kb) [file 12859_2019_2748_MOESM1_ESM.docx]

**Additional file 1. Supplementary Materials**

Materials and Methods

*Technical challenges in modelling of the OncoTrack Data*

The core data architecture used by both i2b2 and TranSMART is to organise data around a data format that describes data records using a *(Patient, Concept, Value)* format. This i2b2/TranSMART data architecture has one important data integrity constraint: Each (Patient, Concept) pair must be unique. For the OncoTrack data, an ideal tree may look like Fig. S1 left panel, under the constraints imposed by multiple treatments, however, it is more likely to be constructed as Fig. S1 right panel:

As the second concept tree on the right of the Scheme shows, trying to coerce a multi-dimensional data set into a purely patient centric data structure, while technically achievable, forces a cognitive burden on the user, which significantly reduces the advantages of using a knowledge management platform.

*Data curation for loading into tranSMART DB*

Initial data curation with regard to used terminology, data standards, internal item references as well as meta data annotations was conducted via the OncoTrack DB. The following data transformations were implemented in the OncoTrack DB as an automatic data exporting function to streamline the periodic update of data in tranSMART:

Subject and sample information: Subject and sample information are modelled as so-called “low-dimensional data” in tranSMART. For low-dimensional data, each variable is shown as a leaf node in the tranSMART-i2b2 data tree. The low dimensional data currently available in OncoTrack for the different subject types involved in the research (patients, *in vivo*, *in vitro* and *in silico* models) have been curated into separate (sub-)studies for each subject type, together with reference data (i.e. mapping to parent subject level) which capture the interrelationships between the subjects across different (sub-)studies.

Drug responses in xenograft, 3D cell cultures and *in silico* experiments: Drug response data are stored in tranSMART with corresponding IDs (treatment group, model, parent sample, parent patient etc.) and the administered compounds for all experiments. In the xenograft subtree, the tumor volume (treatment) over control volume value (T/C), as well as the relative tumor volumes (RTV) are stored. In the 3D cell cultures and *in silico* subtrees, half maximal inhibitory concentration (IC_50_) are stored. For 3D cell cultures the statistics of inhibitory concentration (Mean-Min, Mean-Max, SD-Min, SD-Max) are stored.

Small Genomic Variants: Small genomic variants detected by DNA sequencing for somatic mutations for all sequenced tumour samples are merged to one VCF file as an input for the tranSMART VCF extract, transform, loading (ETL) scripts. Sample identification numbers and their parent patient mapping information (IDs) are also stored in the same subtree.

Transcriptome data: RNA sequencing data are stored as Reads Per Kilobase of transcript per Million mapped reads (RPKM) for each sample. Reads are mapped to their corresponding Ensembl ID as annotations. Sample and their parent patient mapping information (IDs) are also stored in the same subtree.

Epigenome data: The methylation data has been stored in tranSMART as the difference of beta-values between each tumour sample and the average control sample for each gene/gene feature. The original dataset contained approximately 485,000 probes. We have filtered out probes that fail in at least one extract, probes on ChrX and ChrY, and non-CG positions. After this filtering, approximately 426,000 probes remain in the dataset. As a second step, the dataset is split into regions representing a unique gene and gene feature. The probe space is then reduced to about 180,000 regions. Next, regional methylation scores for each extract (and control panel mean) are calculated. A delta-beta is then calculated as in formula (1). Delta-beta < 0 indicates hypomethylated in the Tumour extracts; Delta-beta > 0 represents hypermethylated in the Tumour extracts. For each region, the number of probes from which the mean is calculated is presented.

Delta-beta= Beta_Tumour – Average (Beta_Control)           (1)

Non-coding RNA data: miRNA sequencing data are stored as Reads Per Million (RPM) for each sample. Reads are mapped to their corresponding Rfam ID as annotations. Samples and their parent patient mapping information (IDs) are also stored in the same subtree.


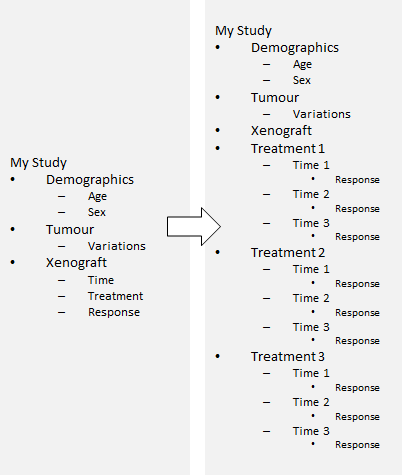


Fig. S1. Problems in representing OncoTrack data in tranSMART/i2b2 data tree.
